# Dentofacial Orthopaedics in Cleft Lip and Palate: A Comprehensive Review

**DOI:** 10.7759/cureus.115236

**Published:** 2026-08-26

**Authors:** Jaweria Shaikh, Rashtra Bhushan, Rakesh Koul, Ram Autar, Swagat Verma

**Affiliations:** 1 Department of Orthodontics and Dentofacial Orthopaedics, Career Post Graduate Institute of Dental Science & Hospital, Lucknow, IND

**Keywords:** alveolar bone grafting, cleft lip and palate, dentofacial orthopaedics, distraction osteogenesis, maxillary protraction, nasoalveolar molding, presurgical infant orthopaedics

## Abstract

Cleft lip and palate (CLP) is a congenital craniofacial anomaly characterised by disruption of the maxilla's form and growth, which involves both a defect in the bony structure and soft tissue that needs multidisciplinary rehabilitation. Dentofacial orthopaedic procedures are extraoral and intraoral forces used to move bone segments and shape and mould future development of the maxillofacial region from the neonatal stage to skeletal maturity. This narrative review summarises current orthopaedic techniques that are used before primary surgery, such as passive maxillary plates, active pin-retained appliances, and nasoalveolar moulding; during deciduous and mixed dentition stages, such as rapid maxillary expansion, protraction facemask therapy, and alternate rapid maxillary expansion and constriction; prior to and following alveolar bone grafting; and prior to and following orthognathic surgery in patients with established maxillary hypoplasia. Clinical evidence is critically evaluated with particular focus on treatment effectiveness, limits of treatment, skeletal and soft tissue outcomes, and clinical application in various stages of cleft rehabilitation. Presurgical orthopaedic procedures usually improve early nasolabial and alveolar form and may aid in surgical management, but it is unclear if skeletal effects are durable and if the skull shape of one procedure is more effective than the other. New technology, such as digitally designed and three-dimensional-printed moulding appliances, temporary skeletal anchorage for maxillary protraction, distraction, and tissue-engineering techniques, is increasing therapeutic options for patients with CLP. Randomised trials with well-designed multicentre randomised trials, standardised outcome measures, and longer follow-up are needed to assess the effectiveness of treatment and facilitate individual orthopaedic management in the future.

## Introduction and background

Orofacial clefts are the most prevalent craniofacial birth defects, occurring in 1.7 per 1,000 live births and showing a strong ethnic and geographic variation. The aetiology includes many susceptibility genes and environmental and epigenetic factors, all of which interfere with the fusion of the facial processes from the 6th to 10th week in the womb [1]. It has an increasing global burden, particularly in low- and middle-income countries, where availability of multidisciplinary care is restricted [2]. The clinical problem does not just affect the lip and soft tissues of the palate. The complete cleft through the maxillary alveolus and into the underlying skeletal foundation results in the displacement and often the collapse of alveolar segments as well as a transverse deficiency of the maxillary arch, distortion of the nasal cartilages, and a further reduction in growth potential due to the scarring that occurs in the primary surgical repair [3]. This dentofacial deformity progresses as a person grows up and usually reaches its peak during childhood with varying degrees of maxillary hypoplasia and Class III skeletal tendency. Rehabilitation demands the coordinated and progressive contributions from a cleft team (surgeon, orthodontist, speech and language therapist, paediatrician, and others) throughout the developmental period of the face [4]. Within the multidisciplinary cleft care team, the orthodontist manages both the dentoalveolar component through orthodontic treatment and the skeletal component through dentofacial orthopaedics, using controlled forces to reposition osseous segments and guide or redirect maxillofacial growth [5]. The neonatal alveolus, the developing mid-face, and the alveolar defect requiring bone grafting are all treated orthopaedically and are all part of a process that interacts with surgical decision-making throughout [6]. The present review presents the concepts of dentofacial orthopaedics in cleft lip and palate (CLP), critically evaluates the clinical evidence available for each of the modalities, and explores technological and scientific advances that are expected to impact future practice.

## Review

Biological reasons and the time frame of intervention

Two properties of the growing craniofacial skeleton are exploited in the cleft patient. The first is the reactivity of the circummaxillary and midpalatal sutures to prolonged mechanical stress, thus allowing for both transverse expansion and forward orthopaedic movement of the maxilla to be maintained as long as the sutures are patent. The second is the transient plasticity of neonatal cartilage and alveolar bone. During the first weeks of life, high levels of oestrogen in circulation from the mother increase hyaluronic acid content while decreasing cartilage elasticity, allowing the production of an improved shape by applying easy and well-controlled pressure to the nasal tissue and alveolar bone [6]. Both are time-limited, and thus, the timing of intervention is as important as the mechanics of intervention.

To conceptualise orthopaedic care in four developmental stages is helpful. The first relates to the neonatal presurgical period, which includes passive and active infant orthopaedics and nasoalveolar moulding prior to primary lip-palate repair. The second is the deciduous dentition, with early transverse or sagittal issues possibly being intercepted at times. The third is the mixed dentition (preparation for and orthodontics for the secondary alveolar grafting, along with maxillary expansion and protraction). The fourth is permanent dentition and skeletal maturity, when the definitive orthodontic alignment is achieved, and any residual skeletal discrepancy is eliminated by distraction osteogenesis (DO) or orthognathic surgery. The sections below are arranged in the same order [7].

Presurgical infant orthopaedics: passive and active appliances

The concept of presurgical infant orthopaedics (PSIO) was first introduced by McNeil in the 1950s, when it was believed that a passive intraoral plate could help reposition the segments of the cleft alveolus into a more favourable relationship, help seal the cleft for feeding, and improve the tongue posture and arch form prior to surgery [8]. The passive maxillary plate, later popularised as the Hotz plate, is not a dynamic device to move segments but rather a device that allows the spontaneous growth of these segments by selective relief and loading of the acrylic, which is still widely used, especially in European centres, mainly as a feeding and arch-guidance aid and not as a definitive orthopaedic device.

A more interventionist approach is taken by active appliances. The pin-retained Latham appliance is an intraoral appliance with an active mechanism (screw or elastic chain) to bring the clefted segments into rapid approximation and realignment and, in bilateral clefts, to retract the protruding premaxilla over a few weeks. The active approach has proved to be effective in decreasing cleft width and thus aiding in surgical closure, but concerns have always been raised that strong early manipulation of the alveolus, particularly in conjunction with gingivoperiosteoplasty, may impede midfacial growth; the long-term effect on the skeleton of the Latham appliance remains uncertain and is a subject of debate [9].

Nasoalveolar moulding

In 1999, Grayson and colleagues introduced nasoalveolar moulding (NAM) as a paradigm shift in PSIO, which included moulding the nasal cartilages and, in bilateral clefts, the missing columella. A custom acrylic intraoral moulding plate gradually brings together the alveolar segments, and one or two acrylic nasal stents help the lower lateral cartilages to assume a more projected and symmetrical shape as well as to elongate the columella during the neonatal period of maximum cartilage malleability [10]. Treatment is started in the first weeks of life and continued, with weekly changes, until primary lip repair is performed at the age of 3-5 months [11].

The great advantage of NAM is that it corrects the nasal deformity (historically the most challenging part to correct secondarily) before the primary surgery, which could increase the aesthetic outcome of the procedure and lead to a decrease in the necessity for revision surgery. Reported improvements of nasal symmetry and columella length; a decrease of alveolar and lip gap, which reduces tension at repair; and, in some series, a decrease in the need for subsequent alveolar grafting in the case of combination NAM and primary gingivoperiosteoplasty (GPP) [12]. The benefits are balanced against an onerous treatment regimen; clinic attendance is often frequent, and parental adherence to treatment is both critical and recognised to be problematic, with mucosal and cutaneous complications.

Presurgical infant orthopaedics: clinical evidence

The best available evidence for passive infant orthopaedics comes from the prospective three-centre randomised controlled trial (Dutchcleft) that compared the passive maxillary plate in infants with complete unilateral CLP and no maxillary plate in the first year of life. In the trial, it has been demonstrated that orthopaedics in the infant period had no clinically relevant effects on the dimensions of the maxillary arch, and there was no prevention of long-term collapse of alveolar segments for the orthopaedics provided in infant orthopaedics [13]. It did not provide any feeding benefit, weight, or length [14], and the transverse dental arch relation at 9 and 12 years of age did not change between the treated and untreated children [15,16]. Considered overall, this evidence does not support the standard use of passive infant orthopaedics based on long-term skeletal and/or occlusal benefit but rather supports feeding or parental or surgical preference.

The evidence base for NAM is greater, but the methods are not as good. The majority of studies that support this are retrospective or short- to medium-term prospective cohorts with good nasolabial results and a lower number of grafts [10,12]. Abbott and Meara noted that the scientific evidence for the efficacy of NAM was not as strong as suggested by its common use due to the lack of good controlled data, the diversity of protocols and outcome measures, and the inability to differentiate the contribution of moulding from the surgeon and the primary repair [17]. There are limited data on whether the early nasal gains extend through the adolescent years, and research into the burden, cost, and benefit continues. The current consensus is that NAM can have long-term skeletal and aesthetic advantages in the hands of an experienced surgeon but that these advantages need to be confirmed by adequately powered randomised trials with standardised outcomes.

The deciduous and mixed dentition ages ranged from 5 to 12 years. The ages of deciduous and mixed dentition were between 5 and 12 years. After the primary dentition is fixed in place, the typical features of the repaired cleft (transverse maxillary constriction, anterior/posterior crossbite, rotation or collapse of the lesser segment) are visible. Rapid or slow maxillary expansion using tooth-borne or bone-borne devices will correct transverse discrepancies, and sometimes it is necessary to expand the segments differently in the cleft maxilla. Also, in the cleft maxilla, expansion is often required to provide access and arch form prior to secondary alveolar bone grafting [18,19].

Orthopaedic protraction is used to treat sagittal maxillary deficiency in the growing child. Cleft patients will have forward skeletal movement when a well-anchored maxilla is treated with a protraction face mask, and Tindlund [20] showed a good skeletal response to maxillary protraction performed prior to the age of 10. The scarred cleft maxilla is often resistant to the conventional protraction, so Liou introduced the protocol of alternate rapid maxillary expansion and constriction (Alt-RAMEC), in which repeated weekly cycles of expansion and constriction loosen the circummaxillary sutures before protraction so as to increase the orthopaedic response, effectively mimicking DO of the sutures [21]. Maxillary advancement using Alt-RAMEC with a face mask or intraoral protraction has been reported to be more effective than maxillary advancement alone in inducing maxillary advancement, with the degree of advantage and its stability varying between studies.

Around secondarily placed alveolar bone grafting (ABG)

Secondary alveolar bone grafting restores continuity of the alveolar ridge, provides bony support for the eruption and orthodontic movement of teeth adjacent to the cleft, and facilitates closure of residual alveolar defects. Bergland and colleagues reported that grafting performed before canine eruption produced the most favourable alveolar bone outcomes and facilitated subsequent orthodontic treatment [22]. The orthodontist typically stretches the collapsed arch and will position the segments into the correct position prior to the grafting to enhance the surgery and arch shape and then will vault the segments back into the newly formed bone after the grafting has consolidated to complete arch integration. When presurgical orthopaedics and primary gingivoperiosteoplasty have already created the bony bridging, secondary grafting might be necessary, but this is one of the most debated aspects of the discipline [23].

Found the hypoplasia of the maxilla in the adolescent and adult

Even with the best of management during the earlier stages of development, there is a significant number of patients with complete CLP who will still have residual hypoplasia of the maxilla, a concave profile, and a negative overjet beyond what orthodontic camouflage can achieve. If the advancement required is moderate, conventional Le Fort I osteotomy can be employed. If the advancement required is large, or if there is extensive scarring and a high risk of relapse, maxillary distraction osteogenesis can be used to allow for gradual advancement along with a concomitant expansion of the overlying soft-tissue envelope, potentially improving the stability of the surgery [24]. There is only one small randomised trial, and the evidence available in this area of surgery is very low quality; neither technique can be declared superior at this time. The decision of which to use should be based on the size of the advancement required, soft tissue considerations, patient age, and operator experience [24]. Maxillary protraction using skeletal anchorage with miniplates or temporary anchorage devices (TADs) is a growing technique in late mixed and early permanent dentition with the goal of achieving a more purely skeletal response with fewer dentoalveolar side effects than a tooth-borne maxillary protraction.

The quality of the evidence base for the conclusions drawn

An over-arching theme of dentofacial orthopaedics in CLP is the lack of confidence in clinical practice compared to the level of supporting evidence. Apart from the Dutchcleft trial, most of the literature is made up of retrospective cohorts and case series with different protocols, short follow-up periods and different outcome measures, which are not consistently defined and hinder pooled analysis. Surgical technique can vary widely from one centre to another and is often not controlled, and the Eurocleft and Scandcleft collaborations demonstrated that the surgical technique was a significant, and often unvariable, confounder of growth outcomes not related to orthopaedic input. Randomised trials should be conducted in several centres; the outcome measures should be standardised and validated; and the results should be reported in a transparent way using cleft registries in order to achieve meaningful progress [25].

Future perspectives

The most obvious technological change is the digitisation of PSIO. Moulding plates can be designed in computer-aided design software, and a complete set of moulding plates can be fabricated using three-dimensional printing in advance, which allows the alveolar and nasal correction to be provided in a planned incremental manner instead of repeated manual correction at the chairside. Digital NAM has the potential to reduce chairside time, minimise patient appointments, enhance consistency between operators, and facilitate remote or 'tele-supervised' care, particularly important in high-burden, resource-limited environments where most clefts are found [26].

In addition to digital moulding, there are other developments that will impact practice. The use of skeletal anchorage using miniplates and TADs is broadening the indications and predictable results of maxillary protraction and can minimise the need for subsequent orthognathic surgery in selected patients [27]. Biotechnology approaches to alveolar defects, such as recombinant human bone morphogenetic protein and cell and/or scaffold-based grafts, are developed to minimise donor site morbidity and enhance graft outcomes. New methods of three-dimensional imaging, finite-element modelling and, more recently, machine learning prediction of growth provide the promise of personalised, biomechanically guided treatment planning. The true value of such innovations will be dependent on rigorous evaluation, in the same strong trial and registry contexts as outlined above, such that innovation is matched with evidence [28].

## Conclusions

Dentofacial orthopaedics as a discipline is woven into the care of patients with CLP, from presurgical moulding of the neonatal alveolus and nose through the growth stage of transverse expansion and maxillary protraction, orthodontic preparation for alveolar bone grafting, and management of established maxillary hypoplasia at skeletal maturity. There is a known biological basis for each modality, and all can be used in care that is holistic and multi-modal. The level of evidence available, however, does not always correspond to the level of clinical use; randomised evidence is limited, while much of the literature on PSIO, nasoalveolar moulding, maxillary protraction, and DO is observational or lower level. PSIO can help to correct early nasolabial and alveolar deformity and help the surgeon plan their approach to the surgery; however, the long-term skeletal results are not well established. In growing patients, maxillary protraction (with or without alternate rapid maxillary expansion and constriction) may be a beneficial procedure to improve maxillary relationships; however, this effect is not always significant or stable. There is relatively little evidence to compare DO with conventional orthognathic surgery. The use of digitally designed and manufactured moulding appliances, skeletal anchorage, and tissue engineering is promising but must be carefully evaluated prior to clinical use. Multicentre randomised studies with long-term, clinically relevant, and standardised outcomes are required to enhance the evidence and provide a greater level of predictability to dentofacial orthopaedic treatment in CLP. However, the level of certainty regarding the effectiveness of these techniques remains variable, highlighting the need for cautious clinical application and individualised treatment planning.
